# Enhanced Anticancer Efficacy of Dual Drug-Loaded Self-Assembled Nanostructured Lipid Carriers Mediated by pH-Responsive Folic Acid and Human-Derived Cell Penetrating Peptide dNP2

**DOI:** 10.3390/pharmaceutics13050600

**Published:** 2021-04-22

**Authors:** Zhe Ma, Jiaxin Pi, Ying Zhang, Huan Qin, Bing Zhang, Nan Li, Zheng Li, Zhidong Liu

**Affiliations:** 1 State Key Laboratory of Component-Based Chinese Medicine, Tianjin University of Traditional Chinese Medicine, Tianjin 301617, China; 18233181543@163.com (Z.M.); pijiaxin@tjutcm.edu.cn (J.P.); zhangying120@hotmail.com (Y.Z.); doxnana169@163.com (H.Q.); zhangbing2018@gmail.com (B.Z.); linan20080402@163.com (N.L.); 2 Engineering Research Center of Modern Chinese Medicine Discovery and Preparation Technique, Ministry of Education, Tianjin University of Traditional Chinese Medicine, Tianjin 301617, China; 3 Institute of Traditional Chinese Medicine, Tianjin University of Traditional Chinese Medicine, Tianjin 301617, China; 4 College of Pharmaceutical Engineering of Traditional Chinese Medicine, Tianjin University of Traditional Chinese Medicine, Tianjin 301617, China

**Keywords:** breast cancer, gambogic acid, paclitaxel, cell penetrating peptide, pH sensitive folic acid

## Abstract

The poor ability of recognition and penetration of chemotherapeutic agents to tumor cells are still great challenges for targeted breast cancer treatment. Herein, we established a tumor-targeted nanostructured lipid carrier encapsulating gambogic acid (GA) and paclitaxel (PTX), which was co-modified with acid-cleavable folic acid (cFA) and a human-derived cell penetrating peptide dNP2 (CKIKKVKKKGRKKIKKVKKKGRK). The multi-functional nano-platform exhibited an enhanced targeting and penetrability to tumor tissues, which was accomplished by the combined action of cFA and dNP2. After intravenous injection, firstly, cFA could actively target the breast cancer tissues by the selective recognition of folate receptor (FR); then, upon arrival at the tumor microenvironment, the acid-cleavable FA and dNP2 dual modified nanostructured lipid carrier (cFA/dNP2-GA/PTX-NLC) exhibited sensitive cleavage of folic acid (FA), which could reduce the hindrance effect of FA to maximize the dNP2 cell-penetrating properties. The effect of different modification on cellular uptake, in vivo bio-distribution, and anticancer activity of NLCs proved our hypothesis that compared with NLCs modified by non-cleavable FA or a single ligand, cFA/dNP2-GA/PTX-NLC displayed more efficient intracellular delivery, stronger targeting ability in vivo, improved cytotoxicity on 4T1 cells, and produced the better therapeutic efficacy of GA and PTX. The strategy affords a feasible way to overcome the poor recognition and permeability of medicines in cancer treatment.

## 1. Introduction

Cancer is the world’s leading cause of death [1], especially in China [2]. As one of the most common malignant tumors among women, breast cancer has the highest incidence and mortality among females, which increases rapidly the global health care burden [1]. Chemotherapy, as a main therapeutic approach of cancer treatment, can kill tumor cells effectively. However, due to the lack of targeting and specificity, it often acts on normal tissues and cells, resulting in low drug efficacy and significant side effects [3]. Furthermore the metastatic spread of cancer cells and multidrug resistance (MDR) are also currently hindering the breast cancer successful therapy [4]. MDR is considered to be one of the main causes of chemotherapy failure. This is because the cancer patients often develop resistance to single chemotherapy drugs, which leads to the decline of the subsequent curative effect [5]. Meanwhile, there were evidence to prove that the overwhelming majority of cancer deaths are due to tumor invasion and metastasis [6]. The combination of different anti-tumor active ingredients has become the preferred scheme of tumor therapy, exhibiting promising antitumor potential and an important complementary and synergistic role. Our previous studies revealed that the combination of GA and PTX has the multi-functional clinical efficacy of three birds with one stone. GA can not only reverse the MDR of PTX by inhibiting the activity of P-glycoprotein in MCF-7/ADR cells but also inhibit the migration and invasion of MDA-MB-231 cells, which plays a crucial role in the prevention and treatment of PTX induced lung metastasis of breast cancer. The synergistic anti-tumor efficiency of GA and PTX was also demonstrated in vivo and in vitro [7]. Therefore, GA and PTX, as a multi-drug combination therapy, provided novel insights for the treatment of breast cancer exerting a better therapeutic efficiency.

As the second generation of a new lipid nano-carrier system, nanostructured lipid carriers (NLCs) have excellent biocompatibility compared with polymer-based nano carriers [8]. Meanwhile, NLCs are characterized by excellent drug-loading capacity, higher drug incorporation rate, sustained release, and better stabilization properties owing to the mixture of liquid and solid lipids [9], and they inherit the superiority of slow drug release and the long resident time in blood from traditional nanoparticles. Therefore, NLCs exhibit an indispensable role in the treatment of diseases, including cancer [10].

Cell-penetrating peptides (CPPs) can be cationic, amphiphilic, or hydrophobic, consisting of 30 or fewer amino acid [11]. CPPs can effectively penetrate cell membranes and enter cells without causing toxic reactions, which attracts the attention of researchers a great deal. The CPP-modified drug delivery system can penetrate into tumor tissues and cells and significantly enhance the anti-tumor effect of drugs [12]. dNP2 (CKIKKVKKKGRKKIKKVKKKGRK) peptide, a human-derived CPP with the potential to delivery drug into nucleus, has been shown to be superior to CPPs TAT and R9 in promoting cell uptake [13,14,15], has less toxicity compared with RRRRRRRR (R8, a classic CPP), and shows the potential of clinical application in cancer treatment [15]. Over the past decade, CPPs have been widely used in tumor delivery of nano-carriers due to their overwhelming ability of cell internalization and penetration. However, the non-selectivity of CPPs in vivo greatly limits the application of CPPs-mediated drug vectors [16]. Folate receptor (FR) is highly expressed in many tumor cells and in up to 80% of breast cancer tumors [17], while normal tissue and cells have lower or negligible expression levels of FR, which makes folic acid (FA) an excellent tumor-targeting moiety [18,19]. FA, which binds to FR with high affinity and is internalized by receptor-mediated endocytosis, could be used for the specific targeting of breast cancer.

Therefore, the dual modification of FA and dNP2 may show good targeting and penetrability to tumor tissues of breast cancer. In the tumor microenvironment (TME), FA could target the breast cancer cells by the specific binding FR. Nevertheless, for cell uptake, CPPs-mediated cell penetration is more effective than receptor-mediated endocytosis by the previous reports [20]. So, the function of dNP2 may be affected by the steric hindrance of FA, which may weaken the cell-penetrating properties of dNP2. Considering the lower pH value of TME compared with normal tissue, the penetrability function of dNP2 can be maximized by conjugating FA with NLCs by acid-cleavable hydrazone bonds, which also guaranteed the targeted effect of FA. Upon arrival at the TME, the acid cleavage of the FA ligand and the exposure of dNP2 peptide contribute to the internalization of the tumor cells of the NLCs, ensuring the NLCs to penetrate deeply into the tumor tissue [21,22].

Based on this idea, we developed acid cleavage FA and dNP2 peptide co-modified NLCs (cFA/dNP2-GA/PTX-NLC) for the efficient delivery of GA and PTX into breast cancer cells, so as to achieve the therapeutic effect of the multifunctionality of GA and PTX.

## 2. Materials and Methods

### 2.1. Materials, Cell Lines, and Animals

PTX with a purity of more than 98% was acquired from Shanghai yuanye Bio-Technology Co., Ltd. (Shanghai, China); GA with a purity of more than 98% was purchased from Sichuan Weikeqi Bio-Tech Co. Ltd. (Chengdu, China); Compritol 888 ATO (Glycerol behenate) was obtained from Gattefossé (St. Priest, France); Myrj 52 (Polyethylene Glycol (40) Monostearate) was obtained from Saint-Priest Cedex, France; MCT 812 (Miglyol^®^ 812) was purchased from Beijing Feng Li Jing Qi Trading Co., Ltd. China; LIPOID S-100 (Soybean Lecithin) was obtained from Shanghai Dong Shang Industrial Co., Ltd., China; DSPE-PEG_2K_-dNP2 (Supplementary Figure S1) were synthesized by Chinapeptide Biotech Co. Ltd. (Shanghai, China); DSPE-Hyd-PEG_5K_-FA and DSPE-PEG_5K_-FA were synthesized by Xi’an Ruixi Biological Technology Co., Ltd. (Xi’an, China); Coumarin-6 (Cou-6) was acquired from Sigma (Saint Louis, MO, USA); 1,10-dioctadecyl-3,3,3,3-tetramethyl indotricarbocyanine iodide (DiR) was obtained from Biotium Inc. (Hayward, CA, USA); 0.25% trypsin + 0.02% EDTA, double antibiotics (10000U Penicillin streptomycin) and Fetal Bovine Serum (FBS) were acquired from Gibco; Cell Counting Kit 8 (CCK-8) was obtained from Dojindo Laboratories (Kumamoto, Japan); PBS, RMPI-1640 medium and 96-well white basal cell culture plate were obtained from Corning, NY; Hoechst 33342 fluorescent probes were acquired from Sigma-Aldrich (Saint Louis, MO, USA); Dulbecco’s modified eagle medium (DMEM) was acquired from Gibco (Gibco, NY, USA). All the other chemicals with analytical reagent grades were acquired from Tianjin Guangfu Fine Chemical Research Institute (Tianjin, China).

MDA-MB-231 cells, 4T1 cells, and MCF7 cells were acquired from ATCC (Manassas, VA, USA), cultivated in RPMI-1640 medium (89%) including 1% penicillin–streptomycin (100 mg/mL streptomycin, 100 units/mL penicillin), and 10% FBS in a humidified atmosphere containing 5% CO_2_ at 37 °C. Human pulmonary adenocarcinoma A549 cells were purchased from ATCC (Manassas, VA, USA), cultivated in DMEM (89%, Gibco) including 1% penicillin–streptomycin and 10% FBS.

BALB/c nude mice (female, 18–22 g) were purchased from Beijing huafukang biotechnology co., LTD and housed in a specific pathogen-free (SPF) environment with access to water and food ad libitum. Animals were used for the experiments after a weeklong acclimation period. All animal experiments were carried out according to the protocol, which was approved by the Ethics Committee of Tianjin University of traditional Chinese medicine (Ethics Code: TCM-LAEC2020024).

### 2.2. Preparation of NLC

The emulsification and solvent evaporation method was used to prepare NLCs as before [7,23]. To get the NLCs modified by different materials, such as FA, dNP2, FA/dNP2, and cFA/dNP2, the soybean lecithin was replaced using an equivalent quantity of the formulated materials, which was determined by the reported methods [24]. GA and PTX (GA and PTX were selected to be encapsulated in NLCs at a calculated mass ratio of 1.18:1 by the previous research results [7]), fluorescent dye Cou-6, and DiR was added to the oil phase when preparing GA/PTX-NLC, Cou-6-NLC, or DiR-NLC.

### 2.3. Particle Size, Zeta Potential, and Morphology

Zetasizer, Nano ZS (Malvern Instruments, Malvern, UK) was used to determine the particle size, polydispersity index (PDI), and zeta potential (ZP) of the unmodified and modified GA/PTX-NLC. Transmission electron microscopy (TEM, JEOL, Akishima, Japan) was used to measure the particle morphology of the unmodified and modified GA/PTX-NLC.

### 2.4. Entrapment Efficiency and Drug Loading

The ultrafiltration method and a validated ultra performance liquid chromatography (UPLC) method [7] were used to determine the encapsulation efficacy (EE) and drug-loading capacity (DL) of GA and PTX in NLC.

The EE (%) calculation formula is as follows:

$$\mathrm{EE}\;\left(\%\right)\;=\;\left(1-\frac{Wfree}{Wtotal}\right)\times 100\%$$

where *Wtotal* and *Wfree* are the total weight of GA and PTX in the NLC and the weight of free GA and PTX, respectively.

The DL (%) calculation formula is as follows:

$$\mathrm{DL}\;\left(\%\right)=\left[\frac{\left(\mathrm{PTX}+\mathrm{GA}\right)Encapsulated}{\left(\mathrm{Lipid}\right)Total}\right]\times 100\%$$

where (Lipid)*Total* and (PTX + GA)*Encapsulated* are the total lipid weight of NLC and the total weight of GA and PTX in the NLC.

### 2.5. Differential Scanning Calorimetry

The physical state of GA and PTX inside the different formulations (modified and unmodified NLC) was investigated by differential scanning calorimetry (DSC). DSC measurements were conducted in a thermal analyzer (DSC822e, Mettler Toledo GmbH, Greifensee, Switzerland). The different samples were heated from 30 to 250 °C with a constant heating rate of 10 °C/min under a 50 mL/min nitrogen flow.

### 2.6. In Vitro Stability

The unmodified and modified GA/PTX-NLC were prepared in triplicate, stored in a refrigerator at 4 °C, and protected from light. Samples were taken at 0, 5, 10, and 15 days after the start, the appearance of the preparation was observed, and the particle size and distribution, the ZP, the EE, and the DL were measured to evaluate its stability.

The stability of the unmodified and modified NLCs in the absence or presence of FBS was measured using a multi-functional microplate reader. The operation was as follows: the unmodified and modified NLCs were mixed with the same volume of FBS, oscillated slowly at 30 RPM, and incubated at 37 °C. At 0, 2, 4, 6, 8, 12, 24, and 48 h after incubation, 200 μL of the mixture of NLCs and FBS was placed in a 96-well plate, and each well transmittance was measured at 750 nm.

### 2.7. Cellular Uptake

Uptake of the unmodified and modified NLCs by 4T1 cells (FR-positive) [25], MDA-MB-231 cells (FR-positive) [26], MCF7 cells (low level FR expression) [26,27], and A549 cells (FR-negative) [28] were measured by the Operetta HCA system (PerkinElmer, MA, USA) using Cou-6 as a fluorescent probe. The cellular uptake of the unmodified and modified NLCs were studied at non-toxic concentration to ensure the carrier and fluorescent dye will not affect the activity of cells.

4T1 cells, MDA-MB-231 cells, and MCF7 cells were seeded in 96-well flat clear-bottom black-wall microplates; after incubation for 24 h, the previous medium was replaced with fresh cell culture medium containing Cou-6-Sol, Cou-6-NLC, FA-Cou-6-NLC, dNP2-Cou-6-NLC, FA/dNP2-Cou-6-NLC, and cFA/dNP2-Cou-6-NLC with an equivalent concentration of Cou-6 at pH 7.4 for 4 h, 8 h. To evaluate the pH-cleavable FA, which could reduce the hindrance effect of FA to maximize the dNP2 cell-penetrating properties, the above cells were also incubated with cFA/dNP2-Cou-6-NLC for 4 h and 8 h at pH 6.8. Then, 50 μL RMPI-1640 medium containing Hoechst 33342 fluorescent probes (with a concentration of 1.67 μg/mL) was added. Cells were incubated for 30 min at 37 °C in the dark. The fluorescent images were acquired in each confocal scan. Columbus (PerkinElmer) was used to calculate the mean fluorescence intensity values of each image.

To investigate the competitive inhibition of FA-Cou-6-NLC uptake via FR-mediated endocytosis, the 4T1 cells, MDA-MB-231 cells, and MCF7 cells were first incubated with free FA (1 mM) to block FR binding and then further treated with FA-Cou-6-NLC nanoparticles for 4 h and 8 h. In addition, the A549 cells (FR-negative) were also used in cell uptake studies.

### 2.8. In Vivo Bio-Distribution Study

The tumor targeting and dynamic distribution of different NLC formulations in BALB/c nude mice with 4T1 cells were studied using DiR as a fluorescence probe by an In Vivo Imaging System (IVIS). Briefly, the female BALB/c nude mice were subcutaneously injected with 0.1 mL of 4T1 cell suspension with a cell density of 2 × 10^7^ cells/mL. The mice were randomly divided into seven groups (a: saline, b: DiR-Sol, c: DiR-NLC, d: dNP2-DiR-NLC, e: FA-DiR-NLC, f: FA/dNP2-DiR-6-NLC, and g: cFA/dNP2-DiR-NLC; n = 6), while the tumor volume of mice grew up to 200 mm^3^ (tumor volume V = ab^2^/2; a: tumor length, b: tumor width). The different NLC formulations were administered at a dose of 0.5 mg/kg of DiR. At 4, 8, 24, and 36 h after administration, mice were anesthetized with 4% chloral hydrate and then placed in an IVIS to observe the fluorescence intensity of each organ with excitation wavelength of 710 nm and emission wavelength of 790 nm. After 24 h, three mice in each group were sacrificed; the heart, lung, liver, spleen, kidney, and tumor were collected, and then, the fluorescence intensities of them were measured.

### 2.9. Cytotoxicity Study

A CCK-8 kit was used to measure the cytotoxicity of different NLC formulations. 4T1 cells were seeded onto 96-well culture plates with a density of 0.8 × 10^4^ cells/well and incubated for 24 h. GA/PTX-Sol, the unmodified and modified GA/PTX-NLC (GA concentration 0.4 μmol/L, PTX concentration 0.25 μmol/L) were added and incubated for 24 h (pH 7.4). In addition, cells were treated with cFA/dNP2-GA/PTX-NLC at pH 6.8 to test whether the cytotoxicity of GA/PTX-NLC was promoted by the acid-cleavable FA. After incubation for 24 h, the previous culture medium was removed. Then, 100 μL 10% CCK-8 diluent was added and incubated for 3 h at 37 °C. A multi-functional microplate reader (MultiskanMK3; Thermo Scientific, Atlanta, GA, USA) was used to measure the absorbance of 450 nm wavelength. We used the following formula to calculate the cell inhibition rate (CIR, %).

$$\mathrm{Cell}\;\mathrm{inhibition}\;\mathrm{rate}\;\left(\%\right)=\left(1-\frac{\mathrm{As}-\mathrm{Ab}}{\mathrm{Ac}-\mathrm{Ab}}\right)\times 100\%$$

where A_c_, A_s_, and A_b_ refer to the control wells, the experimental wells, and the absorbance of the blank wells, respectively.

### 2.10. Caspase Activity Assay

The Caspase-Glo^®^ assay kit (Promega, Madison, WI, USA) was conducted to measure the caspase-3/7 activity by the manufacturer’s instructions. First, 4T1 cells were seeded in 96-well microplates and incubated for 24 h. Then, GA/PTX-Sol, the unmodified and modified GA/PTX-NLC (GA concentration 0.4 μmol/L, PTX concentration 0.25 μmol/L) were added and incubated for 24 h at pH 7.4. In addition, 4T1 cells were also treated with cFA/dNP2-GA/PTX-NLC at pH 6.8. Afterwards, the caspase-3/7 assay reagent of 100 μL was added into each well and incubated for 1 h in the dark. A microplate reader (SpectraMax M5) was used to measure the luminescence of each well. Caspase activity was expressed as fold of the untreated control.

### 2.11. Statistical Analysis

All values were represented as mean ± standard deviation (SD). IBM SPSS Statistics 22.0 software was used to analyze the statistical significance with the *p* value < 0.05 or *p* value < 0.01 or *p* value < 0.001 indicating significance.

## 3. Results and Discussion

### 3.1. Particle Size, Zeta Potential, and Morphology

**Figure 1:**
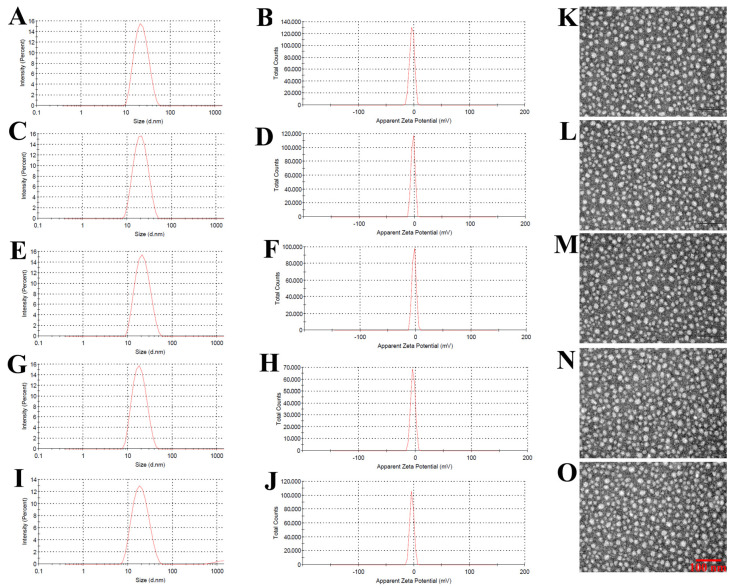
The size and morphology of the formulations: (**A**–**J**) are particle size distribution and zeta potential of GA/PTX-NLC, dNP2-GA/PTX-NLC, FA-GA/PTX-NLC, FA/dNP2-GA/PTX-NLC, and cFA/dNP2-GA/PTX-NLC; as determined by dynamic light scattering. (**K**–**O**) represents the TEM images of them, respectively (scale bar = 100 nm).

The sizes of the unmodified and modified GA/PTX-NLC were approximately 20 nm with PDI ≤ 0.25, and the ZP ranges −2.78 to −4.01 mV (as shown in Table 1), which suggested an acceptable particle size distribution of NLCs. The size distribution of NLCs is suitable for the targeted therapy of breast cancer [7]. The typical particle size distribution and ZP for the different GA/PTX-NLC are shown in Figure 1A–J. The morphological characteristics of the different NLCs were evaluated using TEM. The morphological results showed that all of the NLCs tested in this research are spherical with a uniform particle size (Figure 1K–O).

### 3.2. Entrapment Efficiency and Drug Loading

The EE of GA and PTX are shown in Table 1, all of which were greater than 95%; and the above-mentioned EE indicated that the NLC has good encapsulation capability for GA and PTX, which also may be attributed to the high lipid solubility of PTX and GA. There was no significant difference in DL and EE between modified and unmodified GA/PTX-NLC, indicating that the EE and DL of GA/PTX-NLC not affected by surface modification.

### 3.3. Differential Scanning Calorimetry

**Figure 2:**
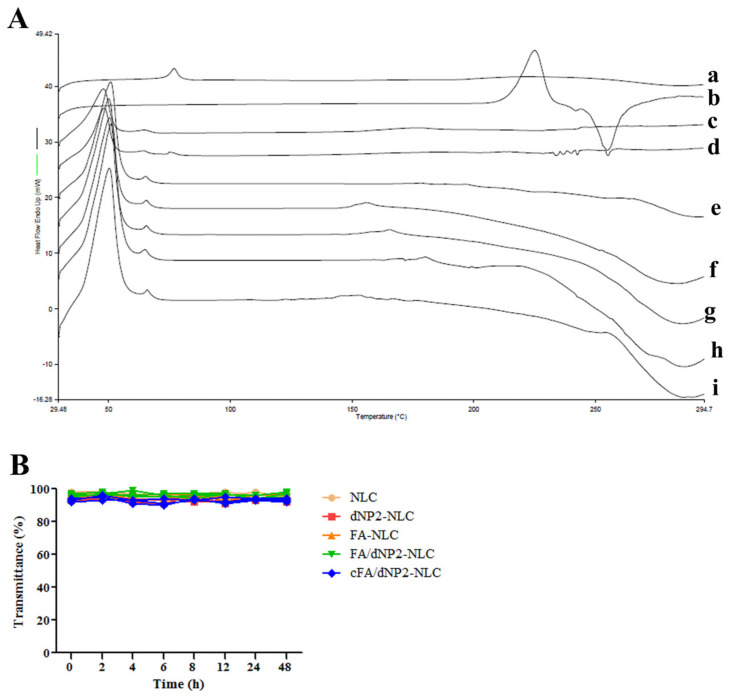
(**A**) Differential scanning calorimetry (DSC) of different formulations (a: GA, b: PTX, c: Blank-NLC, d: the physical mixture of NLC’s formulation and GA + PTX, e: GA/PTX-NLC, f: dNP2-GA/PTX-NLC, g: FA-GA/PTX-NLC, h: FA/dNP2-GA/PTX-NLC, i: cFA/dNP2-GA/PTX-NLC). (**B**) The variations of transmittance of NLCs in 50% Fetal Bovine Serum (FBS) over 48 h (mean ± SD, n = 3).

DSC is widely used to explore the lattice changes of drugs in mixed systems. The DSC thermograms of GA, PTX, Blank-NLC, GA/PTX and the physical mixture of NLC’s formulation, the unmodified and modified GA/PTX-NLC are shown in Figure 2A. By the DSC study, the thermogram revealed that the GA melting peak was 76.54 °C, PTX was 255.07 °C, and both of the above melting peaks were also detected in the physical mixture of NLC’s formulation and GA/PTX. However, the melting peak of GA and PTX were not observed in all NLC formations (the melting peak of the NLC formations appeared at 50.45 °C and 65.87 °C). This may indicate that GA and PTX were successfully encapsulated in amorphous form, GA and PTX did not leak from the nano-carrier system [29].

### 3.4. In Vitro Stability

The long-term stability of NLC in different conditions is essential to ensure the quality of the formulation [30]. The particle size, ZP, EE, and DL of NLCs (stored at 4 °C) were monitored for 15 days to study the physical storage stability. There was no formation of aggregates observed in the unmodified and modified GA/PTX-NLC stored at 4 °C within 15 days. The particle size of the modified GA/PTX-NLC showed a slight increment, while the ZP slightly decreased. The EE and DL of NLCs have not changed significantly within 15 days (Table S1). Therefore, NLCs suspensions were relatively stable after half a month when stored at 4 °C.

After incubation of NLC with FBS for 48 h, there was no aggregation (as shown in Figure 2B), which indicated that the unmodified and modified GA/PTX-NLC had satisfactory serum stability.

### 3.5. Cellular Uptake Study

We investigated the endocytosis of different modified Cou-6-NLCs in 4T1, MDA-MB-231, MCF7, and A549 cells to evaluate the uptake efficiency of different tumor cells to cFA/dNP2-Cou-6-NLC. Cou-6 was selected for cell uptake study with an appropriate concentration (0.05 μg/mL) as described previously [31].

**Figure 3:**
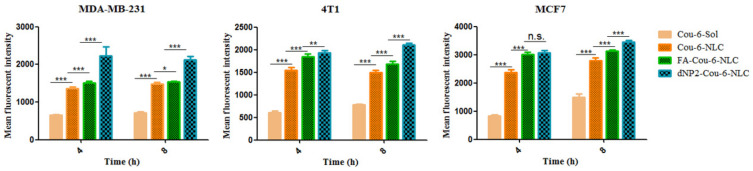
The cellular uptake in MDA-MB-231, 4T1, and MCF-7 cells after treatment with Cou-6-Sol, Cou-6 labeled NLC, and modified Cou-6 labeled NLC at different incubation time intervals (4 h, 8 h). * *p* < 0.05; ** *p* < 0.01; *** *p* < 0.001; n.s., not significant. Results are expressed as mean ± SD, n = 3.

**Figure 4:**
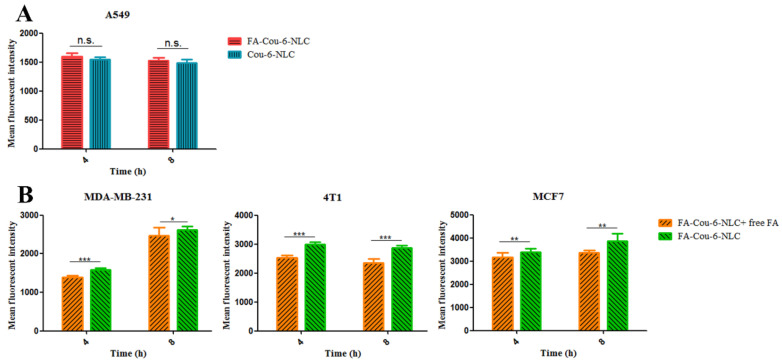
(**A**) The cellular fluorescence intensity of Cou-6-NLC and FA modified Cou-6-NLC at different incubation time intervals (4 h, 8 h) in A549 cells. (**B**) Blocking experiment after pretreatment with excess FA. * *p* < 0.05; ** *p* < 0.01; *** *p* < 0.001; n.s., not significant. Results are expressed as mean ± SD, n = 3.

**Figure 5:**
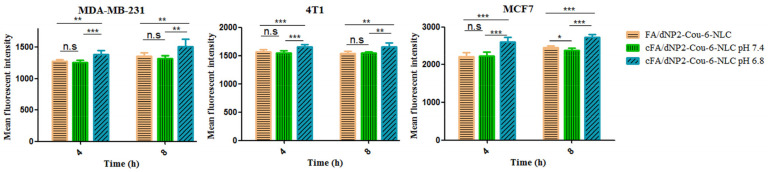
The cellular uptake in MDA-MB-231, 4T1, and MCF-7 cells after treatment with Cou-6-loaded different modified NLCs. * *p* < 0.05; ** *p* < 0.01; *** *p* < 0.001; n.s., not significant. Results are expressed as mean ± SD, n = 3.

The cell uptake of various Cou-6-loaded NLCs and Cou-6-Sol using equivalent Cou-6 concentrations is shown in Figure 3, Figure 4 and Figure 5. In 4T1, MDA-MB-231, and MCF7 cells, after incubation for 4 h and 8 h, the intake of the dNP2-Cou-6-NLC and FA-Cou-6-NLC exhibited a significantly higher uptake than Cou-6-NLC and solution, namely the fluorescent intensity: modified Cou-6-NLC > Cou-6-NLC > Cou-6-Sol (Figure 3), suggesting the lower internalization ability of Cou-6-Sol. The ability of modified NLC to recognize cancer cells was greatly improved compared with unmodified NLC. The cell uptake of dNP2 decorated NLCs were greater than that of FA-modified NLCs (Figure 3), suggesting that the cellular uptake mediated by CPP was much more efficient than receptor-mediated endocytosis, which is consistent with the previous reports [20].

The cellular uptake was also evaluated in A549 cells using Cou-6-NLC and FA- Cou-6-NLC. The results showed that no significant difference was observed in cell intake between Cou-6-NLC and FA-Cou-6-NLC (Figure 4A), which may be caused by the fact that A549 cells negatively express FR, so FA-Cou-6-NLC entered cells through non-specific endocytosis rather than receptor-mediated endocytosis, which is consistent with the way of Cou-6-NLC entering cells. In addition, in the competitive inhibition experiment, the fluorescence intensity of FA-Cou-6-NLC + free FA (pre-treatment with 1 mM free FA) was significantly decreased compared to FA-Cou-6-NLC (Figure 4B). The cellular uptake of FA-Cou-6-NLC was significantly inhibited by free FA. These results suggested that FA-Cou-6-NLCs were internalized into the MDA-MB-231, 4T1, and MCF7 cells owing to the receptor-mediated endocytosis, and the targeting ability of FA was also further demonstrated.

When cFA is scissored by the lower pH of the TME and activated to dNP2, the cellular uptake of cFA/dNP2-Cou-6-NLC is expected to be enhanced due to the significant penetration effect of dNP2. The 4T1 cells, MDA-MB-231 cells, and MCF7 cells were selected to investigate the uptake of cFA/dNP2-Cou-6-NLCs after lower the pH trigger cleavage. At pH 7.4, non-cleavable and cleavable Cou-6-NLC showed similar internalization, while the internalization ability of cFA/dNP2-Cou-6-NLC (pH 6.8) was significantly higher than that of cFA/dNP2-Cou-6-NLC (pH 7.4) and FA/dNP2-Cou-6-NLC (*p* < 0.01, Figure 5), which suggested that the exposure of dNP2 further promoted the internalization of nano-carriers. These results indicated that cFA/dNP2 could be degraded at the lower pH value of the TME, thus further improving the cellular uptake [22].

### 3.6. In Vivo Bio-Distribution Study

**Figure 6:**
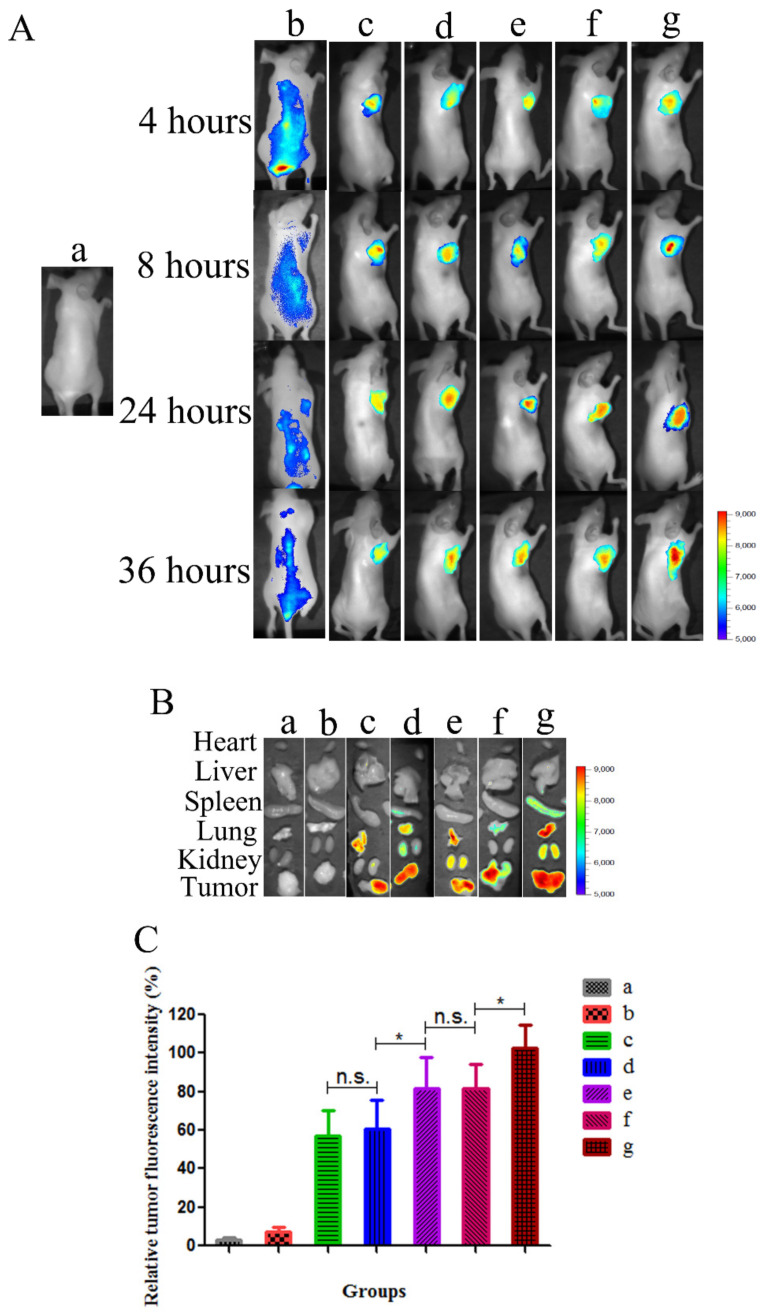
Bio-distribution study of modified DiR-NLC in tumor-bearing mice. (**A**) Fluorescence images of mice treated with different DiR formulations in vivo at different time points (4, 8, 24, and 36 h). (**B**) Ex vivo fluorescence images of the organs and tumor tissues excised from mice treated with different DiR formulations. (**C**) Fluorescence signal of tumor obtained from mice treated with different DiR formulations. Results are expressed as mean ± SD, * *p* < 0.05. n.s., not significant. In Figure 6, a: Sal, b: DiR-Sol, c: DiR-NLC, d: dNP2-DiR-NLC, e: FA-DiR-NLC, f: FA/dNP2-DiR-6-NLC and g: cFA/dNP2-DiR-NLC.

As shown in Figure 6, the mice of the saline group exhibited no significant fluorescence. Due to the non-specific distribution and rapid clearance after intravenous injection, no significant DiR accumulation was observed in the tumor site during the imaging period in the DiR-Sol group. After 4 h, the fluorescence of DiR in the DiR-Sol group decreased gradually. These groups of unmodified and modified DiR-NLC could be clearly observed to have DiR fluorescence in tumor tissues. Among all the formulations, cFA/dNP2-DiR-NLC (g) displayed the strongest fluorescence intensity at 4, 8, 24, and 36 h. This is partly due to the combination of FR-mediated tumor targeting and dNP2 deep penetration. The exposure of dNP2 peptide after the cleavage of FA further elevated the penetration ability of NLCs in tumor tissues. In contrast, the fluorescence intensity of non-cleavable FA/dNP2-DiR-6-NLC (f) is almost the same as FA-DiR-6-NLC (e) due to the steric hindrance of FA. The above results provided evidence in vivo that cFA/dNP2-DiR-NLC increased the accumulation of DiR in tumor cells.

Meanwhile, unlike the cellular uptake results, in which dNP2-DiR-NLC displayed higher internalization than FA-DiR-NLC, the biodistribution in vivo exhibited that FA-DiR-NLC (e) was superior to dNP2-DiR-NLC (d) in tumor targeting (Figure 6C, *p* < 0.05), probably owing to the tumor targeting effect of FA ligand. The cellular uptake results in vitro were consistent with the imaging results in vivo, showing that cFA/dNP2-DiR-NLC was superior to non-cleavable FA/dNP2-DiR-6-NLC and single ligand modified NLCs (Figure 6B,C). The dual modification NLCs of cFA and dNP2 ligand had a higher accumulation in tumor tissues [21].

### 3.7. Cytotoxicity Study

**Figure 7:**
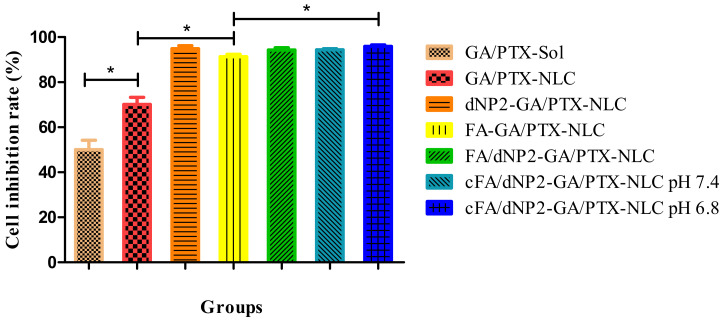
The cell viability of different formulations was analyzed by Cell Counting Kit 8 (CCK-8). Results are expressed as mean ± SD, n = 3. * *p* < 0.05.

The previous results showed that the combination of GA (0.4 μmol/L) and PTX (0.25 μmol/L) exhibited the stronger synergistic anti-tumor efficacy [7]. In this part, the anti-tumor activities of GA/PTX-Sol, the unmodified and modified GA/PTX-NLC were evaluated using the above drug concentration with 4T1 cells after 24 h incubation. As shown in Figure 7, the cytotoxicity of GA and PTX was significantly enhanced when they were encapsulated with NLC, which may be due to the enhanced endocytosis by the tumor cells, and it was consistent with the results of cell uptake. In addition, NLC could improve the stability of GA and PTX.

The cytotoxicity of the unmodified and modified NLCs was also evaluated. The cytotoxicity of FA and/or dNP2 modified NLC were significantly higher than that of the unmodified NLC. The above result may be caused by the following reasons: Binding FA and/or dNP2 to the surface of NLC can enhance uptake by 4T1 cells, thereby increasing the concentration of GA and PTX in the cells, which will help to improve the therapeutic efficiency of tumor. The cytotoxicity of cFA/dNP2-GA/PTX-NLC was higher than FA-GA/PTX-NLC (*p* < 0.05). However, no significant difference was observed in other modified NLCs groups. The possible reason is that NLCs groups already have a high inhibition rate on cancer cells [7].

### 3.8. Caspase Activity Assay

**Figure 8:**
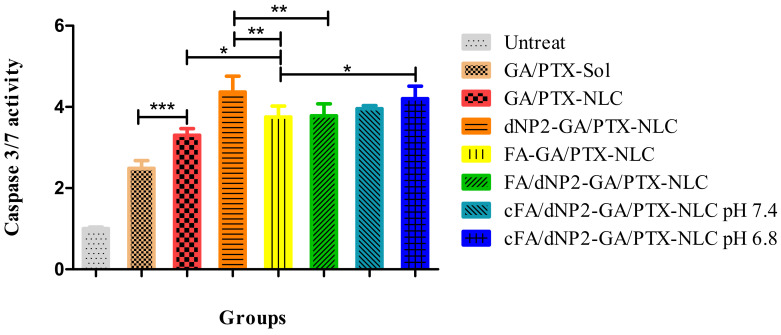
Caspase 3/7 activity in 4T1 cells treated by different formulations. Results are expressed as mean ± SD, n = 3. * *p* < 0.05; ** *p* < 0.01; *** *p* < 0.001.

The results of previous studies have shown that the combination of GA and PTX exhibited more apoptotic cells than the single drug in tumor tissues of mice, and the induction effect of GA and PTX encapsulated with NLC on the apoptotic death of cancer cells was significantly greater than that of solution [7]. As caspases are responsible for the execution of apoptosis, the activation of caspase was detected using a caspase activity kit. As shown in Figure 8, the treatment of GA/PTX-Sol resulted in increased caspase 3/7 activity (2.4-fold to control) in 4T1 cells. When GA and PTX were loaded by NLC, the caspase 3/7 activity (3.3-fold to control) was further increased, and the caspase-3/7 activities of 4T1 cells were increased by 4.3-, 3.7-, 3.7-, 3.9-, and 4.2-fold after 24 h treatment of dNP2-GA/PTX-NLC, FA-GA/PTX-NLC, FA/dNP2-GA/PTX-NLC, cFA/dNP2-GA/PTX-NLC (pH 7.4), and cFA/dNP2-GA/PTX-NLC (pH 6.8), respectively compared with the control group.

Taken together, these results suggested that NLC enhances GA/PTX-induced 4T1 cell apoptosis, and the lower pH value could cleave cFA/dNP2 to form dNP2, which could further enhance cell uptake, thus increasing the concentration of GA and PTX in cells, eventually leading to increased apoptotic cells, which is consistent with the cellular uptake study and cytotoxicity study.

## 4. Conclusions

We reported a multi-functional nano-platform co-modified by acid-cleavable FA ligand and dNP2 peptide to assist the delivery of GA and PTX to breast cancer. The cFA/dNP2 decoration could increase the cellular uptake and cytotoxicity of GA/PTX-NLC in breast cancer cells at pH 6.8. cFA/dNP2-GA/PTX-NLC displayed sensitive cleavage of FA in acidic TME and maximized the function of FA ligand and dNP2 peptide. Compared with NLCs modified by non-cleavable FA/dNP2 or a single ligand, cFA/dNP2-GA/PTX-NLC exhibited higher accumulation in breast cancer in vivo bio-distribution study, induced an enhancement of apoptosis, and achieved the maximal anticancer efficiency of GA and PTX by retaining the synergistic tumor targeting and penetration of FA and dNP2. This multi-target strategy has proven to be effective for targeted breast cancer therapy.

## Figures and Tables

**Table 1:** Particle size, polydispersity index (PDI), zeta potential (ZP), encapsulation efficacy (EE), and drug-loading capacity (DL) of GA/PTX-NLC and targeted GA/PTX-NLC (mean ± SD, n = 3).

Preparation	Size (nm)	PDI	ZP (mV)	EE% (GA)	EE% (PTX)	DL% (GA)	DL% (PTX)
GA/PTX-NLC	23.62 ± 0.36	0.16 ± 0.03	−3.59 ± 1.80	95.13 ± 0.64	96.14 ± 0.23	7.45 ± 0.07	6.43 ± 0.04
dNP2-GA/PTX-NLC	21.28 ± 0.33	0.18 ± 0.06	−2.96 ± 0.43	98.15 ± 0.63	95.18 ± 0.21	7.54 ± 0.06	6.33 ± 0.05
FA-GA/PTX-NLC	21.93 ± 0.58	0.22 ± 0.07	−2.78 ± 0.86	96.07 ± 0.74	96.79 ± 0.49	7.50 ± 0.07	6.47 ± 0.04
FA/dNP2-GA/PTX-NLC	19.38 ± 0.89	0.25 ± 0.09	−4.01 ± 0.26	95.69 ± 0.88	97.17 ± 0.27	7.48 ± 0.05	6.47 ± 0.08
cFA/dNP2-GA/PTX-NLC	20.36 ± 0.78	0.23 ± 0.05	−3.88 ± 0.96	97.36 ± 0.56	95.69 ± 0.58	7.51 ± 0.06	6.39 ± 0.05

## Data Availability

The datasets used and/or analyzed during the current study are available from the corresponding author on reasonable request.

## Supplementary Materials

The following are available online at https://www.mdpi.com/1999-4923/13/5/600/s1, Figure S1: (A) dNP2 (CKIKKVKKKGRKKIKKVKKKGRK) MALDI-TOF REPORT; (B) DSPE-PEG_2K_-dNP2 MALDI-TOF REPORT. Figure S2: The microscopy image of cell uptake of Cou-6 labeled formulations by MDA-MB-231, 4T1, MCF-7 and A549 cells. Table S1: The particle size, PDI, ZP, EE, and DL of GA/PTX-NLC (Day 15).

## Abbreviations

GA: gambogic acid; PTX: paclitaxel; TCMs: traditional Chinese medicines; NLCs: nanostructured lipid carriers; SLN: solid lipid nanoparticle; CPPs: cell penetrating peptides; DiR: 1,10-dioctadecyl-3,3,3,3-tetramethyl indotricarbocyanine iodide; Cou-6: Coumarin-6; PDI: polydispersity index; EE: encapsulation efficacy; DL: drug-loading capacity; FR: folate receptor; FA: folic acid; MDR: multidrug resistance; TME: tumor microenvironment; dNP2: CKIKKVKKKGRKKIKKVKKKGRK; cFA: FA-PEG_5K_-Hyd-DSPE; CCK-8: Cell Counting Kit 8; TEM: transmission electron microscopy; UPLC: ultra performance liquid chromatography; DSC: differential scanning calorimetry; IVIS: In Vivo Imaging System; CIR: cell inhibition rate; ZP: zeta potential.

## References

[1] Bray F., Ferlay J., Soerjomataram I., Siegel R.L., Torre L.A., Jemal A. (2018). Global cancer statistics 2018: GLOBOCAN estimates of incidence and mortality worldwide for 36 cancers in 185 countries. Ca A Cancer J. Clin..

[2] Chen W., Zheng R., Baade P.D., Zhang S., Zeng H., Bray F., Jemal A., Yu X.Q., He J. (2016). Cancer Statistics in China, 2015. Ca A Cancer J. Clin..

[3] Ma Z., Fan Y., Wu Y., Kebebe D., Zhang B., Lu P., Pi J., Liu Z. (2019). Traditional Chinese medicine-combination therapies utilizing nanotechnology-based targeted delivery systems: A new strategy for antitumor treatment. Int. J. Nanomed..

[4] Tian F., Dahmani F.Z., Qiao J., Ni J., Xiong H., Liu T., Zhou J., Yao J. (2018). A targeted nanoplatform co-delivering chemotherapeutic and antiangiogenic drugs as a tool to reverse multidrug resistance in breast cancer. Acta Biomater..

[5] Wang S., Yang Y., Wang Y., Chen M. (2015). Gambogic acid-loaded pH-sensitive mixed micelles for overcoming breast cancer resistance. Int. J. Pharm..

[6] Kreusel K.-M., Bechrakis N.E., Wiegel T., Krause L., Foerster M.H. (2008). Incidence and clinical characteristics of symptomatic choroidal metastasis from lung cancer. Acta Ophthalmol..

[7] Ma Z., Li N., Zhang B., Hui Y., Lu P., Pi J., Liu Z. (2020). Dual drug-loaded nano-platform for targeted cancer therapy: Toward clinical therapeutic efficacy of multifunctionality. J. Nanobiotechnol..

[8] Ma Z., Zhang B., Fan Y., Wang M., Kebebe D., Li J., Liu Z. (2019). Traditional Chinese medicine combined with hepatic targeted drug delivery systems: A new strategy for the treatment of liver diseases. Biomed. Pharmacother..

[9] Üner M. (2006). Preparation, characterization and physico-chemical properties of solid lipid nanoparticles (SLN) and nanostructured lipid carriers (NLC): Their benefits as colloidal drug carrier systems. Die Pharm. Int. J. Pharm. Sci..

[10] Carbone C., Leonardi A., Cupri S., Puglisi G., Pignatello R. (2014). Pharmaceutical and biomedical applications of lipid-based nanocarriers. Pharm. Pat. Anal..

[11] Guidotti G., Brambilla L., Rossi D. (2017). Cell-Penetrating Peptides: From Basic Research to Clinics. Trends Pharmacol. Sci..

[12] Kebebe D., Liu Y., Wu Y., Vilakhamxay M., Liu Z., Li J. (2018). Tumor-targeting delivery of herb-based drugs with cell-penetrating/tumor-targeting peptide-modified nanocarriers. Int. J. Nanomed..

[13] Albanese A., Tang P.S., Chan W.C.W. (2012). The Effect of Nanoparticle Size, Shape, and Surface Chemistry on Biological Systems. Annu. Rev. Biomed. Eng..

[14] Lim S., Kim W.-J., Kim Y.-H., Lee S., Koo J.-H., Lee J.-A., Yoon H., Kim D.-H., Park H.-J., Kim H.-M. (2015). dNP2 is a blood–brain barrier-permeable peptide enabling ctCTLA-4 protein delivery to ameliorate experimental autoimmune encephalomyelitis. Nat. Commun..

[15] Xiang Y., Shan W., Huang Y. (2018). Improved anticancer efficacy of doxorubicin mediated by human-derived cell-penetrating peptide dNP2. Int. J. Pharm..

[16] Liu Y., Mei L., Xu C., Yu Q., Shi K., Zhang L., Wang Y., Zhang Q., Gao H., Zhang Z. (2016). Dual Receptor Recognizing Cell Penetrating Peptide for Selective Targeting, Efficient Intratumoral Diffusion and Synthesized Anti-Glioma Therapy. Theranostics.

[17] Parker N., Turk M.J., Westrick E., Lewis J.D., Low P.S., Leamon C.P. (2005). Folate receptor expression in carcinomas and normal tissues determined by a quantitative radioligand binding assay. Anal. Biochem..

[18] Dosio F., Arpicco S., Stella B., Brusa P., Cattel L. (2009). Folate-mediated targeting of albumin conjugates of paclitaxel obtained through a heterogeneous phase system. Int. J. Pharm..

[19] Xie M., Zhang H., Xu Y., Liu T., Chen S., Wang J., Zhang T. (2013). Expression of folate receptors in nasopharyngeal and laryngeal carcinoma and folate receptor-mediated endocytosis by molecular targeted nanomedicine. Int. J. Nanomed..

[20] Liu Y., Ran R., Chen J., Kuang Q., Tang J., Mei L., Zhang Q., Gao H., Zhang Z., He Q. (2014). Paclitaxel loaded liposomes decorated with a multifunctional tandem peptide for glioma targeting. Biomaterials.

[21] Li M., Shi K., Tang X., Wei J., Cun X., Chen X., Yu Q., Zhang Z., He Q. (2018). pH-sensitive folic acid and dNP2 peptide dual-modified liposome for enhanced targeted chemotherapy of glioma. Eur. J. Pharm. Sci. Off. J. Eur. Fed. Pharm. Sci..

[22] Li M., Shi K., Tang X., Wei J., Cun X., Long Y., Zhang Z., He Q. (2018). Synergistic tumor microenvironment targeting and blood-brain barrier penetration via a pH-responsive dual-ligand strategy for enhanced breast cancer and brain metastasis therapy. Nanomed. Nanotechnol. Biol. Med..

[23] Huang R., Li J., Kebebe D., Wu Y., Zhang B., Liu Z. (2018). Cell penetrating peptides functionalized gambogic acid-nanostructured lipid carrier for cancer treatment. Drug Deliv..

[24] Gao W., Meng T., Shi N., Zhuang H., Yang Z., Qi X. (2015). Targeting and Microenvironment-Responsive Lipid Nanocarrier for the Enhancement of Tumor Cell Recognition and Therapeutic Efficiency. Adv. Health Mater..

[25] Hong J., Sun Z., Li Y., Guo Y., Liao Y., Liu M., Wang X. (2017). Folate-modified Annonaceous acetogenins nanosuspensions and their improved antitumor efficacy. Int. J. Nanomed..

[26] Soe Z.C., Thapa R.K., Ou W., Gautam M., Nguyen H.T., Jin S.G., Ku S.K., Oh K.T., Choi H.-G., Yong C.S. (2018). Folate receptor-mediated celastrol and irinotecan combination delivery using liposomes for effective chemotherapy. Colloids Surf. B Biointerfaces.

[27] Kim S.-H., Lim S.-J., Park J.-S., Lee M.-K. (2008). Folate-tethered emulsion for the target delivery of retinoids to cancer cells. Eur. J. Pharm. Biopharm..

[28] Jain A., Fournier P.G.J., Mendoza-Lavaniegos V., Sengar P., Guerra-Olvera F.M., Iñiguez E., Kretzschmar T.G., Hirata G.A., Juárez P. (2018). Functionalized rare earth-doped nanoparticles for breast cancer nanodiagnostic using fluorescence and CT imaging. J. Nanobiotechnol..

[29] Padhye S.G., Nagarsenker M.S. (2013). Simvastatin Solid Lipid Nanoparticles for Oral Delivery: Formulation Development and In vivo Evaluation. Indian J. Pharm. Sci..

[30] Pereira I., Zielińska A., Ferreira N.R., Silva A.M., Souto E.B. (2018). Optimization of linalool-loaded solid lipid nanoparticles using experimental factorial design and long-term stability studies with a new centrifugal sedimentation method. Int. J. Pharm..

[31] Kebebe D., Wu Y., Zhang B., Yang J., Liu Y., Li X., Ma Z., Lu P., Liu Z., Li J. (2019). Dimeric c(RGD) peptide conjugated nanostructured lipid carriers for efficient delivery of Gambogic acid to breast cancer. Int. J. Nanomed..

