# Supplementary material for: Enhanced Anticancer Efficacy of Dual Drug-Loaded Self-Assembled Nanostructured Lipid Carriers Mediated by pH-Responsive Folic Acid and Human-Derived Cell Penetrating Peptide dNP2
Source: Pharmaceutics. 2021 Apr 22;13(5):600. doi: 10.3390/pharmaceutics13050600 (PMC8143576; doi:10.3390/pharmaceutics13050600)
Supplement: Supplementary file 1 [file pharmaceutics-13-00600-s001.zip › pharmaceutics-1165400-supplementary.pdf]

pharmaceutics

MDPI

Supplement Materials: Enhanced Anticancer Efficacy of Dual Drug-Loaded Self-Assembled Nanostructured Lipid Carriers Mediated by pH-Responsive Folic Acid and Human-Derived Cell Penetrating Peptide dNP2

Zhe Ma, Jiaxin Pi , Ying Zhang , Huan Qin , Bing Zhang , Nan Li , Zheng Li and Zhidong Liu

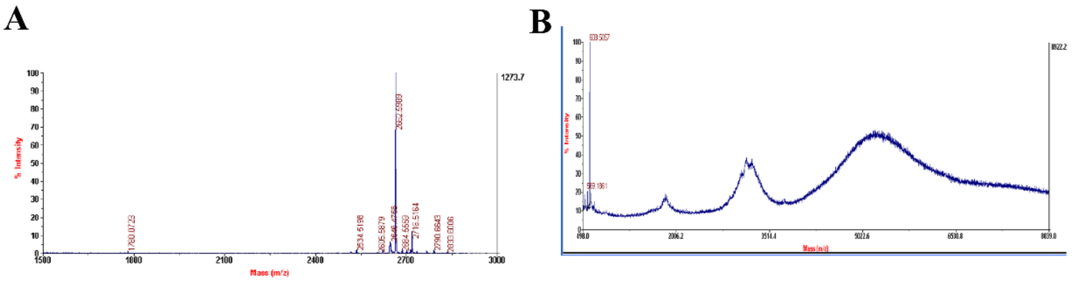

Figure S1. A: dNP2 MALDI-TOF REPORT; B: DSPE-PEG2K-dNP2 MALDI-TOF REPORT.

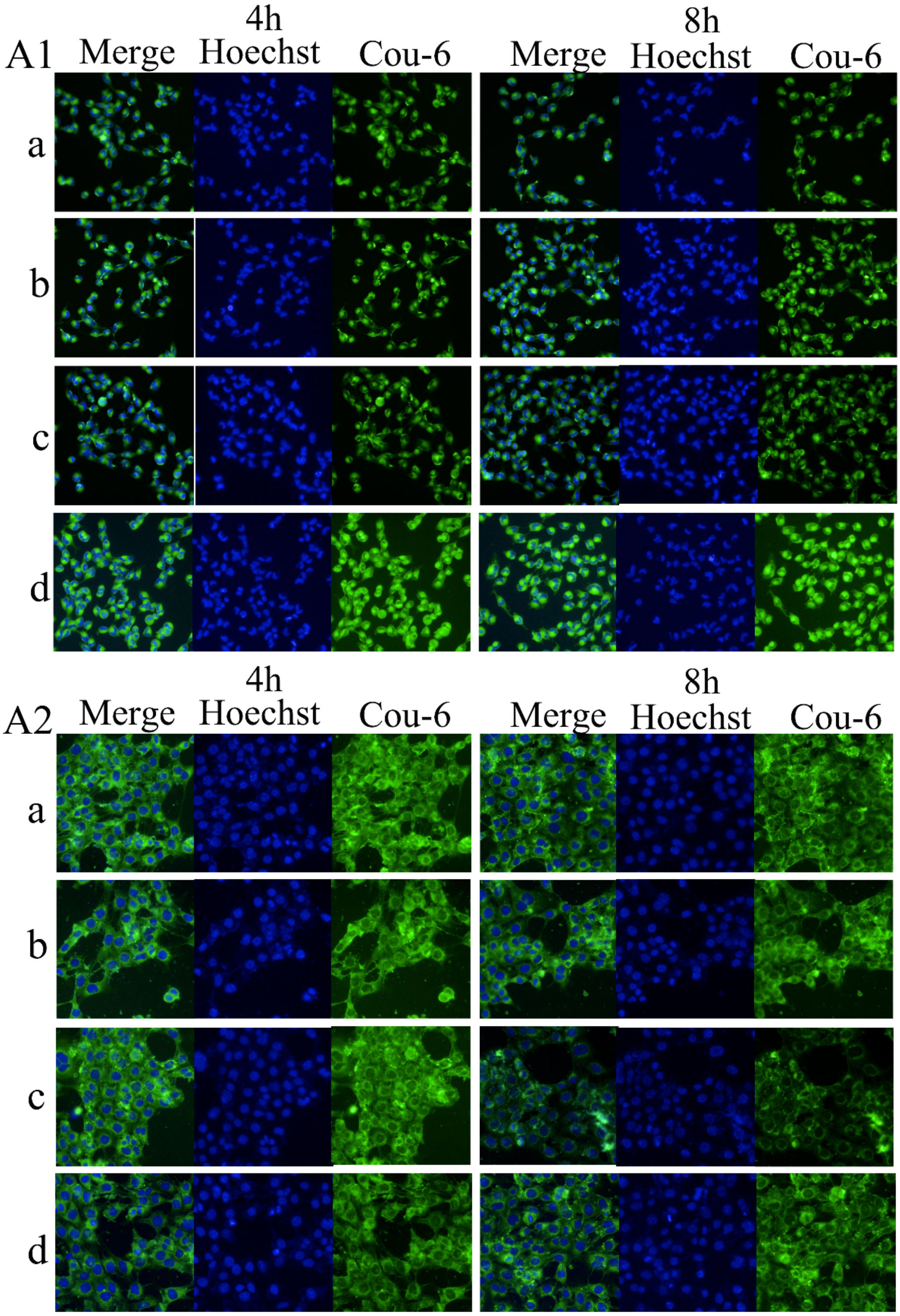
A1
4h
Merge
Hoechst
Cou-6
8h
Merge
Hoechst
Cou-6
a
b
c
d
A2
4h
Merge
Hoechst
Cou-6
8h
Merge
Hoechst
Cou-6
a
b
c
d

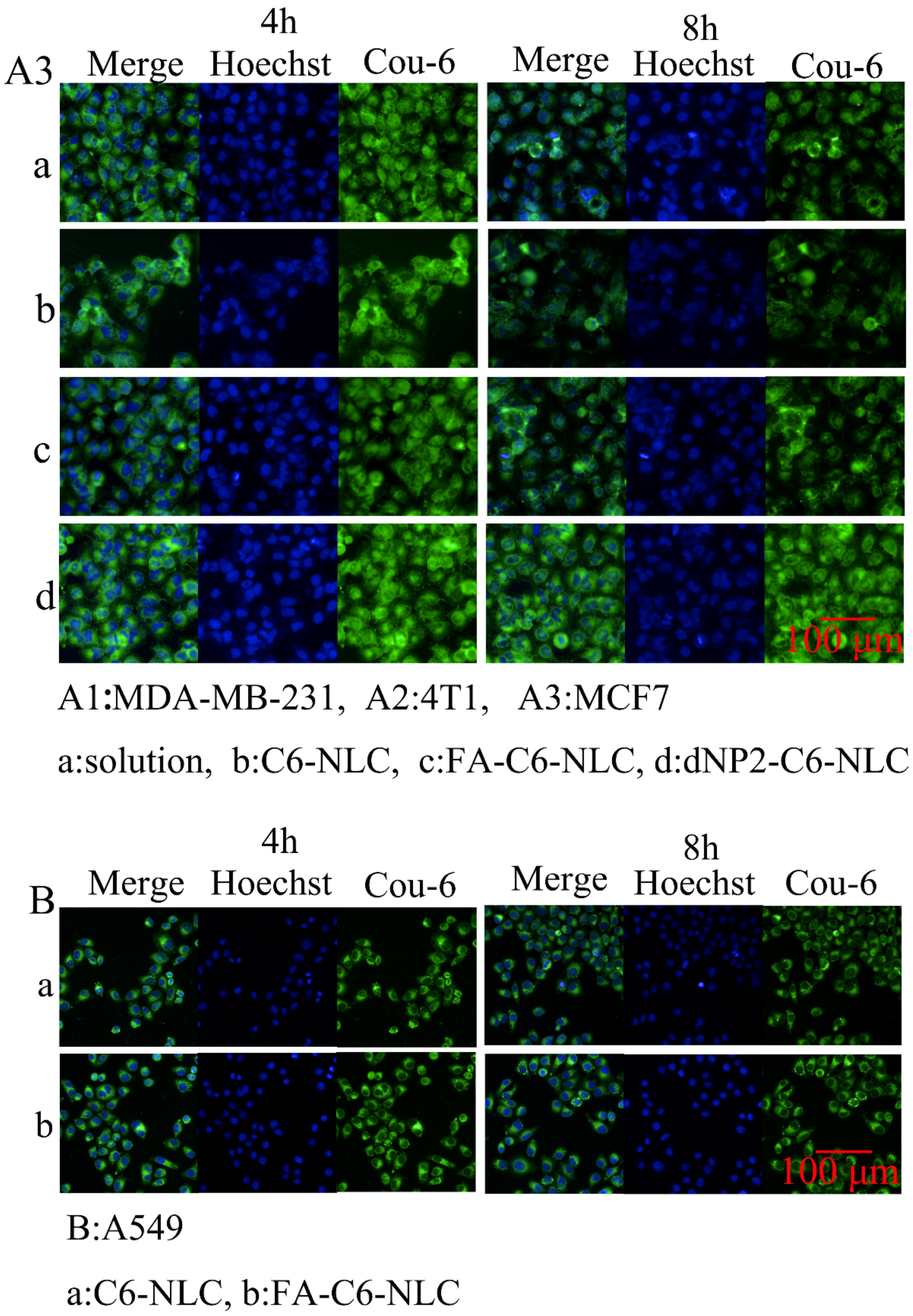
A3
4h
8h
Merge
Hoechst
Cou-6
a
b
c
d
100 μm
A1:MDA-MB-231, A2:4T1, A3:MCF7
a:solution, b:C6-NLC, c:FA-C6-NLC, d:dNP2-C6-NLC
B
4h
8h
Merge
Hoechst
Cou-6
a
b
100 μm
B:A549
a:C6-NLC, b:FA-C6-NLC

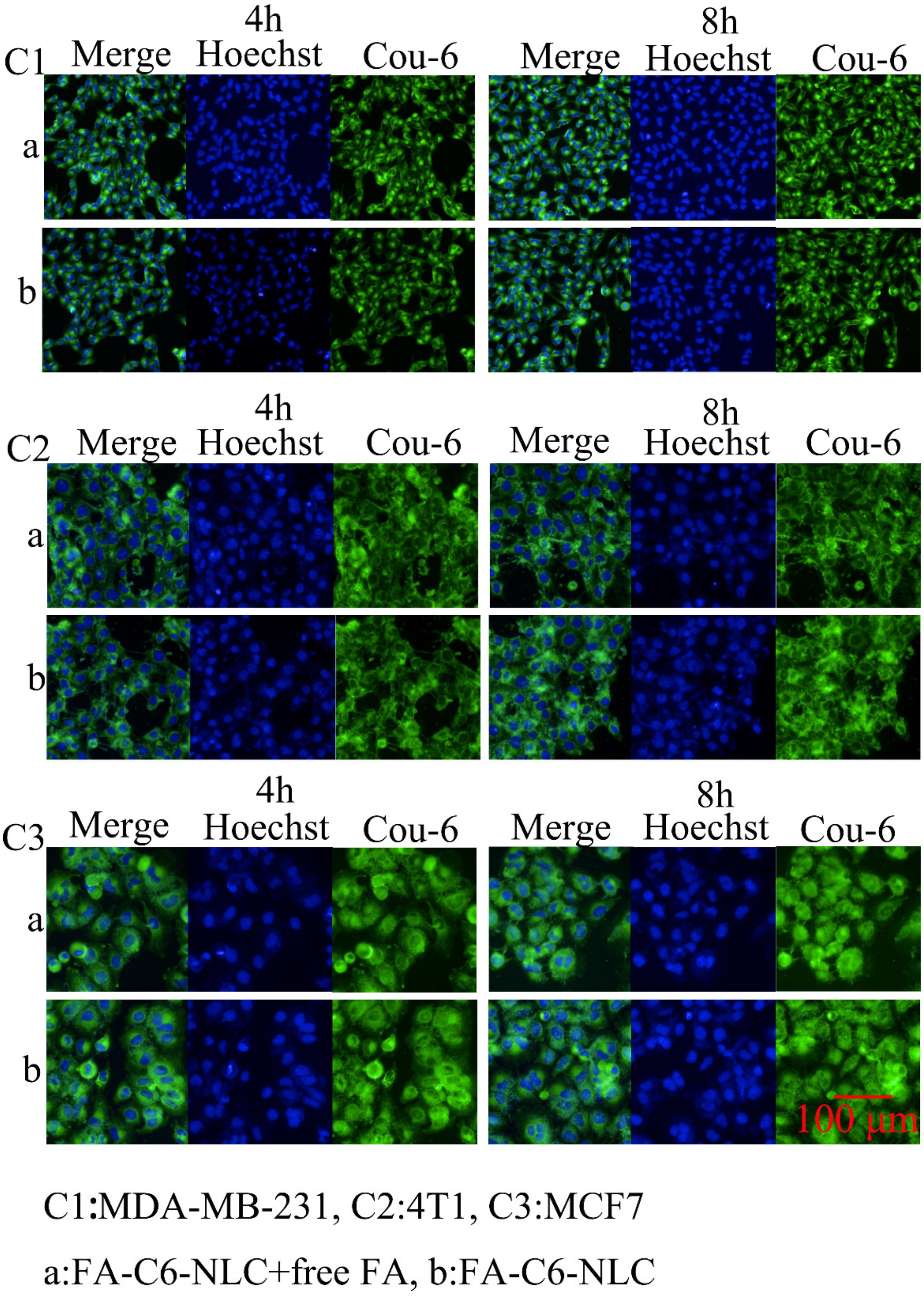
C1
4h
Merge
Hoechst
Cou-6
8h
Merge
Hoechst
Cou-6
a
b
C2
4h
Merge
Hoechst
Cou-6
8h
Merge
Hoechst
Cou-6
a
b
C3
4h
Merge
Hoechst
Cou-6
8h
Merge
Hoechst
Cou-6
a
b
100 μm
C1:MDA-MB-231, C2:4T1, C3:MCF7
a:FA-C6-NLC+free FA, b:FA-C6-NLC

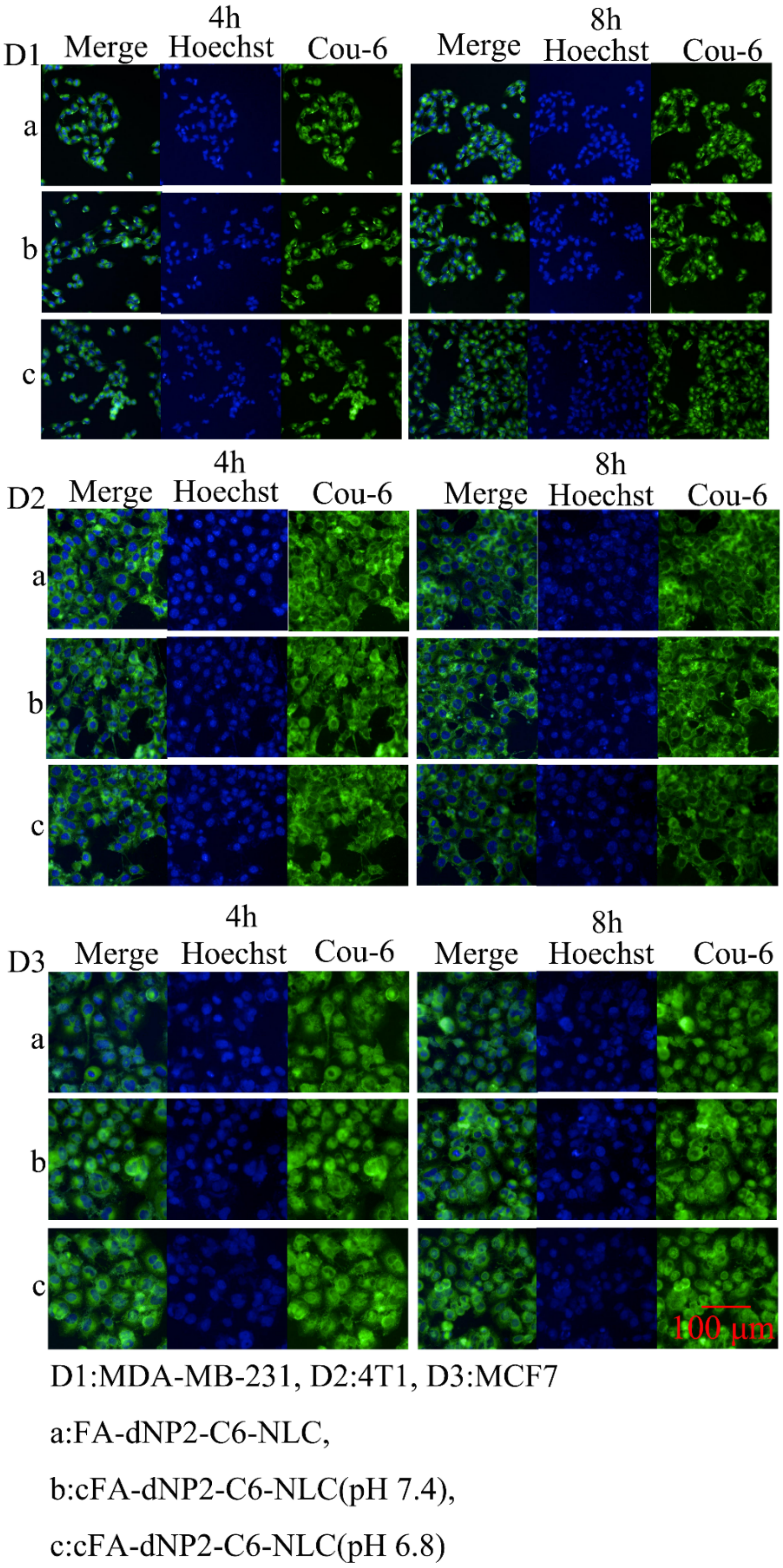

Figure S2. The microscopy image of cell uptake of Cou-6 labeled formulations by MDA-MB-231, 4T1, MCF-7 and A549 cells. A1, C1, and D1: MDA-MB-231 cells; A2, C2, and D2: 4T1 cells; A3, C3, and D3: MCF-7 cells; B: A549 cells (scale bar=100 μm).

Table S1. The particle size, PDI, ZP, EE, and DL of GA/PTX-NLC (Day 15).

Preparation	Size (nm)	PDI	ZP (mV)	EE% (GA)	EE% (PTX)	DL% (GA)	DL% (PTX)
GA/PTX-NLC	32.12	0.39	−4.30	89.64	88.79	6.98	5.89
dNP2-GA/PTX-NLC	33.28	0.41	−5.15	85.13	87.64	6.76	5.14
FA-GA/PTX-NLC	35.29	0.49	−4.76	90.06	86.33	6.99	5.76
FA/dNP2-GA/PTX-NLC	34.13	0.50	−4.61	85.78	90.17	6.53	6.03
cFA/dNP2-GA/PTX-NLC	38.96	0.46	−5.27	91.36	88.69	7.01	5.81
